# Divergent Roles of Inflammation in Skeletal Muscle Recovery From Injury

**DOI:** 10.3389/fphys.2020.00087

**Published:** 2020-02-13

**Authors:** Emily E. Howard, Stefan M. Pasiakos, Christopher N. Blesso, Maya A. Fussell, Nancy R. Rodriguez

**Affiliations:** ^1^Department of Nutritional Sciences, University of Connecticut, Storrs, CT, United States; ^2^Military Nutrition Division, U.S. Army Research Institute of Environmental Medicine, Natick, MA, United States; ^3^Oak Ridge Institute for Science and Education, Oak Ridge, TN, United States

**Keywords:** myogenesis, satellite cells, myogenic regulatory factors, muscle protein turnover, cytokines

## Abstract

A transient increase in local pro-inflammatory cytokine expression following skeletal muscle injury mediates the repair and regeneration of damaged myofibers through myogenesis. Regenerative capacity is diminished and muscle wasting occurs, however, when intramuscular inflammatory signaling is exceedingly high or persists chronically. An excessive and persistent inflammatory response to muscle injury may therefore impair recovery by limiting the repair of damaged tissue and triggering muscle atrophy. The concentration-dependent activation of different downstream signaling pathways by several pro-inflammatory cytokines in cell and animal models support these opposing roles of post-injury inflammation. Understanding these molecular pathways is essential in developing therapeutic strategies to attenuate excessive inflammation and accelerate functional recovery and muscle mass accretion following muscle damage. This is especially relevant given the observation that basal levels of intramuscular inflammation and the inflammatory response to muscle damage are not uniform across all populations, suggesting certain individuals may be more susceptible to an excessive inflammatory response to injury that limits recovery. This narrative review explores the opposing roles of intramuscular inflammation in muscle regeneration and muscle protein turnover. Factors contributing to an exceedingly high inflammatory response to damage and age-related impairments in regenerative capacity are also considered.

## Introduction

Skeletal muscle is the most abundant tissue in the human body, with a vital role in energy and protein metabolism as well as force generation for locomotion and stability. These essential functions can be disrupted with muscle injury occurring as a result of strains, contusions, lacerations, ischemia, burns, or even strenuous exercise. Musculoskeletal injuries requiring surgical interventions may also cause extensive muscle trauma. Damage to the muscle surrounding the hip or knee, for example, is inevitable during total hip or total knee arthroplasty (THA or TKA) procedures, respectively (Agten et al., 2017). Using a tourniquet to maintain a bloodless field during surgery promotes muscle trauma through the associated ischemia and reperfusion injury (Muyskens et al., 2016). Loss of muscle mass or function due to injury may ultimately limit activities of daily living and quality of life.

Damaged skeletal muscle has the intrinsic capacity to regenerate and repair itself through myogenesis. The myogenic response involves activation, proliferation, and differentiation of muscle-resident stem cells (i.e., satellite cells) that ultimately fuse with each other and existing fibers to restore injured tissue (Dumont et al., 2015; Snijders et al., 2015). Muscle regenerative capacity is diminished with advanced age and with chronic inflammation (Suetta et al., 2013; Jin et al., 2018). Intramuscular inflammatory signaling plays a critical role in mediating the regenerative response to muscle fiber damage and must be finely regulated given inflammatory cytokine expression is capable of promoting muscle growth and muscle loss (Dogra et al., 2006, 2007; Munoz-Canoves et al., 2013). A transient increase in local inflammatory signaling triggers a pro-myogenic signaling cascade that aids in the repair, remodeling, and maintenance of healthy muscle tissue. If this intramuscular inflammation persists chronically, however, regenerative capacity is diminished and muscle atrophy ensues. An exceedingly high inflammatory response to muscle damage would limit recovery by preventing the repair of damaged tissue, promoting muscle wasting, and ultimately impairing the restoration of muscle function.

This narrative review explores recent advances delineating the opposing roles of intramuscular inflammation in regeneration, maintenance, and wasting of muscle. Intrinsic and extrinsic factors underlying the loss of regenerative capacity with aging are also discussed. Understanding molecular and cellular processes regulating muscle regeneration versus muscle atrophy downstream of inflammatory signaling, as well as age-related changes in these pathways, is essential in developing therapeutic strategies to attenuate excessive inflammation, restore regenerative capacity, and accelerate recovery following muscle injury. Identifying individuals susceptible to excessive muscle inflammation has also been proposed as a possible method of predicting muscle mass and functional recovery potential following orthopedic injury (Bamman et al., 2015). The utility of this concept in clinical settings is therefore considered.

## Skeletal Muscle Regeneration

### Satellite Cells and Myogenesis

Skeletal muscle regeneration after injury involves the activation of muscle-resident satellite cells located outside the sarcolemma and underneath the basal lamina of muscle fibers (Mauro, 1961). These normally quiescent adult muscle stem cells are regulated by a dynamic interaction of extrinsic factors [i.e., systemic molecules, fibro-adipogenic progenitors (FAP), immune cells] and intracellular signaling pathways that mediate satellite cell quiescence, self-renewal, and the myogenic response (Li, 2003; Girgenrath et al., 2006; Arnold et al., 2007; Fiore et al., 2016). Activated satellite cells generate myoblasts in response to injury-related signals by reentering the cell cycle and proliferating. Myoblasts subsequently differentiate by increasing expression of muscle-specific genes, and eventually fuse with each other or existing myofibers to regenerate and repair damaged tissue. A small subset of activated satellite cells do not commit to myogenic differentiation and instead replenish the satellite cell pool by returning to a quiescent state (Olguin and Olwin, 2004; Zammit et al., 2004).

Satellite cell progression through the myogenic program is driven by the sequential expression of specific transcription factors. Pax7 is a paired box transcription factor that is expressed in all satellite cells of mature muscle and is critical to their function (Seale et al., 2000; von Maltzahn et al., 2013). Terminal differentiation requires down-regulation of Pax7, while elevation of Pax7 subsequent to proliferation promotes a return to quiescence (Olguin and Olwin, 2004; Olguin et al., 2007). Pax7 has also been implicated in satellite cell specification of pluripotent stem cells, as it appears to restrict alternate development programs (Seale et al., 2000). Progression of myogensis also involves Myf5, MyoD, myogenin, and MRF4, transcription factors collectively known as Myogenic Regulatory Factors (MRFs). Myf5 and MyoD are expressed early after injury (Cornelison and Wold, 1997; Cooper et al., 1999), while myogenin and MRF4 have been shown to regulate later stages of myogenic differentiation (Smith et al., 1994; Cornelison and Wold, 1997).

A precise balance of intracellular mechanisms regulating quiescence, differentiation, and self-renewal are necessary to maintain satellite cell function and regenerative capacity. Notch signaling, for example, is critical to multiple cellular processes. Injury-related activation of Notch pathway activity stimulates satellite cell proliferation and inhibits differentiation, allowing for adequate expansion of progenitor cells (Conboy and Rando, 2002). Eventual suppression of this pathway and transition to canonical Wnt signaling appears necessary for proper differentiation (Brack et al., 2008). Notch signaling also plays a role in maintaining satellite cell quiescence and self-renewal capacity. Specific deletion of the Notch signaling protein recombining protein-Jk (RBP-Jk) triggered spontaneous satellite cell activation and attenuated self-renewal, ultimately depleting the satellite cell pool (Bjornson et al., 2012). Low et al. (2018) have also shown that activation of Notch3 by the Notch ligand Delta-like 4 (Dll4) during muscle repair provided a signal for satellite cells to exit the cell cycle and return to quiescence, thereby promoting self-renewal. FOXO3 expression and Sprouty 1 (Spry1) activity have similarly been implicated in the return of satellite cells to quiescence during repair to maintain the satellite cell pool (Shea et al., 2010; Gopinath et al., 2014).

Skeletal muscle autophagy, a cellular process responsible for the degradation of intracellular proteins, macromolecules, and organelles, is critical for skeletal muscle regeneration. Deletion of the autophagy initiation kinase Unc-51-like kinase 1 (Ulk1) in mice subjected to muscle injury impaired recovery of muscle strength and mitochondrial protein content (Call et al., 2017). Age-related declines in autophagic activity are similarly associated with diminished regenerative capacity (Garcia-Prat et al., 2016). Autophagy appears necessary to support rapid changes in metabolic activity and bioenergetic demands of activated versus quiescent satellite cells (Tang and Rando, 2014). Inhibiting autophagy in satellite cells suppressed ATP production and delayed their activation (Tang and Rando, 2014). Myoblast differentiation and fusion capacity were similarly impaired with inhibition of autophagy (McMillan and Quadrilatero, 2014). AMP-activated protein kinase (AMPK)-mediated phosphorylation of p27^Kip1^ and its subsequent translocation from the nuclei to the cytoplasm has been implicated in the regulation of autophagy during regeneration (White et al., 2018). In total, these catabolic mechanisms are imperative to the regenerative process.

### Inflammatory Response to Skeletal Muscle Injury

A highly controlled, time-dependent activation of immune cells occurs rapidly after muscle injury to remove necrotic tissue and release soluble factors that regulate satellite cell activation and progression through the differentiation process (Tidball, 2017; Yang and Hu, 2018). Acute pro-inflammatory signaling and immune cell recruitment to the site of muscle trauma constitutes the initial phase of this inflammatory response. Immune cell infiltration at the site of injury is first stimulated by the complement system, which is activated immediately following muscle damage (Frenette et al., 2000). Injury-related activation of muscle-resident mast cells and their subsequent degranulation and release of inflammatory mediators (i.e., TNFα, IL-1, histamine) similarly promotes immune cell recruitment (Gorospe et al., 1996; Radley and Grounds, 2006). Secretion of the chemoattractants CXC-chemokine ligand 1 (CXCL1) and CC-chemokine ligand 2 (CCL2) as well as damage-associated molecular patterns (DAMPs) such as high-mobility group box 1 protein (HMGB1) from muscle-resident macrophages also facilitates immune cell activation and infiltration following injury (Brigitte et al., 2010; Venereau et al., 2012). Recent work has suggested muscle osteopontin expression is also involved in promoting immune cell infiltration (Wasgewatte Wijesinghe et al., 2019).

Several immune cells are involved in the skeletal muscle regenerative response. Neutrophils are among the first inflammatory cells to infiltrate injured muscle tissue. They are observed as early as 1 – 3 h after muscle damage, with maximum numbers seen 6 – 24 h after injury (Fielding et al., 1993; Malm et al., 2000). These phagocytic cells release proteases and oxidants to facilitate the removal of damaged fibers. Although their presence can aggravate muscle injury (Pizza et al., 2005), neutrophil depletion delays muscle recovery, suggesting their role in clearing cellular debris and recruiting other inflammatory cells is critical for successful regeneration (Teixeira et al., 2003). Recent work also indicates that neutrophils may have anti-inflammatory and inflammation-resolving properties important to the progression of later stages of myogenesis (Sugimoto et al., 2016).

The temporal and spatial recruitment of macrophages is critical to the muscle regenerative response to injury. Neutrophil production of interleukin-1 (IL-1) and interleukin-8 (IL-8) at the site of injury, and the secretion of CCL2 from injured muscle fibers and resident macrophages stimulates the extravasation of monocytes into injured tissue and their subsequent differentiation into macrophages (Fujishima et al., 1993; Lu et al., 2011). Inhibiting this accumulation of monocytes and macrophages in injured muscle using *Ccr2^–/–^* mice and via pharmacological treatment (i.e., liposomal clodronate administration) impairs the regenerative response (Summan et al., 2006; Lu et al., 2011). Autophagy and caspase activation have also been implicated in this monocyte to macrophage transition (Jacquel et al., 2012). Macrophages are initially polarized into a pro-inflammatory M1 phenotype that secretes pro-inflammatory cytokines (i.e., TNFα, IL-1β, IFN-γ) and reactive oxygen species to facilitate the removal of cellular debris and recruitment of immune cells to the lesion site (Villalta et al., 2009; Dort et al., 2019). This early increase in M1 cells precedes the eventual expansion of anti-inflammatory M2 macrophages that repress the local inflammatory response and promote muscle growth. M1 macrophages reach peak numbers 1–2 days after injury, while M2 cells are the predominant macrophage population by 3 days post-injury (Arnold et al., 2007).

Although a mixture of M1 and M2 cells have been observed following injury (Heredia et al., 2013), a switch in the overall bias of macrophage populations from a pro-inflammatory to anti-inflammatory phenotype is critical for the proper progression of the regenerative response. Pro-inflammatory cytokines released from M1 macrophages stimulate myoblast proliferation, while anti-inflammatory cytokines from M2 macrophages promote their differentiation (Arnold et al., 2007). Suppressing this M1 to M2 phenotype transition has been shown to attenuate muscle regeneration and reduce muscle fiber growth (Mounier et al., 2013; Tonkin et al., 2015; Varga et al., 2016). Several molecular processes regulate the ability of macrophages to switch from the M1 to M2 phenotype. Phagocytosis of apoptotic cells by activated macrophages, for example, attenuates macrophage pro-inflammatory cytokine production and enhances their anti-inflammatory activity (Chung et al., 2006). Increased concentrations of IL-10 similarly promote the M2 phenotype (Villalta et al., 2011b), while interferon gamma (IFNγ) signaling attenuates the M1 to M2 conversion (Villalta et al., 2011a). Recent work has also implicated the heme-binding transcriptional repressor BACH1 in this response, as *Bach1* knockout mice displayed impairments in muscle regeneration and the macrophage phenotype switch (Patsalos et al., 2019).

T lymphocyte (T cell) recruitment is also essential in the repair and regeneration of damaged muscle tissue, with infiltration of CD4^+^ and CD8^+^ T cells peaking around 3–5 days post-injury (Fu et al., 2015). T cell-deficient *Rag1*^–/–^ mice have delayed muscle regeneration after injury that is reversed with the transplantation of active CD3^+^ T cells (i.e., general population of activated T cells). Muscle regeneration capacity is similarly attenuated in CD8^+^-deficient mice (Zhang et al., 2014) and with the depletion of Foxp3^+^ CD4^+^ regulatory T cells (T_reg_ cells) (Burzyn et al., 2013). T_reg_ cells appear to regulate muscle regeneration by acting directly on satellite cells and modulating immune cell activity. Accumulation of T_reg_ cells occurs at a time when infiltrating macrophages switch from a M1 pro-inflammatory to M2 pro-regenerative state, and deletion of T_reg_ cells impairs this phenotypic transition and prolongs the inflammatory response (Burzyn et al., 2013). Muscle T_reg_ cells also release amphiregulin, a ligand for the epidermal growth factor receptor that enhances satellite cell differentiation *in vitro* and *in vivo* (Burzyn et al., 2013).

Transient remodeling of extra-cellular matrix (ECM) components and modulation of satellite cell activity by FAP cells is an additional component to the muscle regenerative response to injury. FAPs are stem cells that can differentiate into fibroblasts or adipocytes. They are normally quiescent in healthy muscle, but proliferate rapidly in response to muscle injury (Joe et al., 2010). Depletion of FAPs impairs muscle regeneration after injury and leads to a loss of satellite cells under homeostatic conditions (Wosczyna et al., 2019). FAPs appear to regulate the regenerative response by influencing satellite cell activity. Pharmacological blockage of FAP expansion prevented transient ECM deposition and attenuated myoblast proliferation *in vivo* (Fiore et al., 2016). *In vitro* work similarly demonstrated a dose-dependent increase in myoblast proliferation with the addition of proliferating FAPs to satellite cell cultures (Fiore et al., 2016). This work collectively highlights the critical role of immune cell infiltration, ECM remodeling, and inflammatory signaling in regulating muscle regeneration after injury.

## Potential Consequences of Heightened Intramuscular Inflammatory Signaling

The release of inflammatory cytokines following muscle damage is a finely regulated response. Several pro-inflammatory cytokines (e.g., TNFα, TWEAK) involved in initial stages of muscle regeneration and repair are elevated early in response to damage (Li, 2003; Girgenrath et al., 2006). A shift from pro- to anti-inflammatory signaling (e.g., IL-13, IL-10, IL-4) within days of muscle injury subsequently represses local inflammatory signaling and supports later phases of myogenesis (Arnold et al., 2007; Deng et al., 2012). Heightened basal (pre-injury or pre-surgery) levels of intramuscular inflammation, an exaggerated inflammatory response to muscle damage, or both, would lead to exceedingly high levels of muscle inflammation that may disrupt this finely regulated response. Likewise, failure to resolve persistent pro-inflammatory signaling after muscle injury may dysregulate muscle regeneration and repair.

Exceedingly high or unresolved post-injury inflammation would impact many aspects of the regenerative response. Chronic infection of mouse skeletal muscle (i.e., a preexisting inflammatory environment) delays muscle repair following cardiotoxin injury (Jin et al., 2018). The heightened inflammatory environment of infected muscle appeared to limit macrophage phenotype transition and altered the expression of MRFs following damage (Jin et al., 2018). A delayed switch from pro-inflammatory to pro-regenerative macrophages may also promote fibrosis. An overlap in expression of M1- and M2-derived tumor necrosis factor-α (TNFα) and transforming growth factor beta 1 (TGF-β1), respectively, has been shown to reduce FAP apoptosis and increase ECM deposition (Lemos et al., 2015). Expression of the TNFα receptors TNFR1 and TNFR2 in nerve structures during chronic inflammatory insult in rabbit muscle suggests the consequences of excessive inflammation extend beyond the muscular system (Renstrom et al., 2017).

Persistent pro-inflammatory cytokine signaling may promote muscle wasting by blunting muscle protein synthesis and triggering muscle protein breakdown. This is evident with several pathophysiological conditions of muscle wasting. Burn injury, as an extreme example, leads to skeletal muscle breakdown throughout the body. A marked increase in vastus lateralis mRNA expression of TNFα-, TNF-like weak inducer of apoptosis (TWEAK)- and interleukin (IL)-6-family cytokines and receptors 5-days post burn injury suggests changes in inflammatory signaling may contribute to the observed atrophy (Merritt et al., 2013b). Similarly, elevated expression of TWEAK and TNFα receptors has been observed in paralyzed muscle of men with chronic spinal cord injury, a condition that also presents with muscle atrophy and impaired regenerative capacity (Yarar-Fisher et al., 2016). The opposing regulatory roles of local inflammatory signaling in myogenesis and muscle mass maintenance indicate the post-injury or post-operative inflammatory response must be well-controlled to maintain muscle mass and allow for adequate tissue regeneration. While multiple cytokines are involved in regulating these processes, TWEAK, TNFα, IL-6 and their respective molecular pathways appear to be shared across conditions of both acute and chronic inflammation.

### Divergent Roles of Inflammation in Myogenesis

**FIGURE 1:**
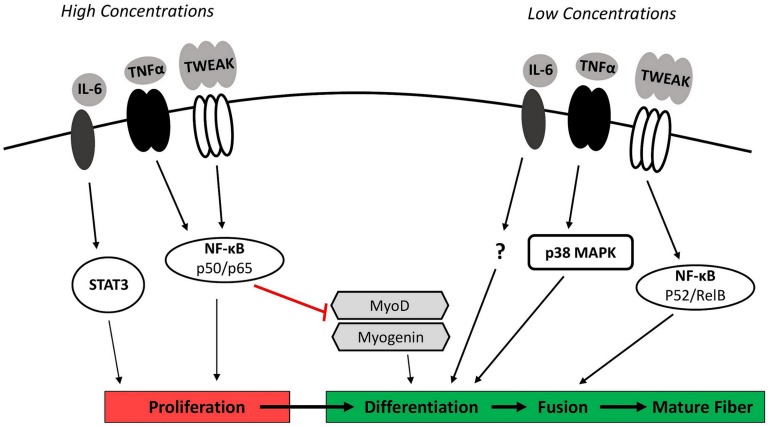
Inflammation and myogenesis. While multiple cytokines are involved in coordinating the myogenic response to injury, major regulators include IL-6, TNFα, and TWEAK. High concentrations of these cytokines signaling via STAT3 and the classical NF-κB pathway (p50/p65 heterodimer) stimulate myoblast proliferation but inhibit subsequent differentiation through the degradation and destabilization of the myogenic regulatory factors MyoD and myogenin. Low concentrations of IL-6, TNFα, and TWEAK signaling through p38MAPK and the alternative NF-κB pathway (p52/RelB heterodimer) are necessary to facilitate later stages of myogenesis by stimulating myoblast differentiation and fusion. IL-6, interleukin-6; NF-κB, nuclear factor-κB; p38 MAPK, p38 mitogen-activated protein kinase; STAT3, signal transducer and activator of transcription 3; TNFα, tumor necrosis factor-α; TWEAK, TNF-like weak inducer of apoptosis.

The opposing roles of inflammation in promoting and inhibiting myogenesis correspond with observed concentration- and time-dependent effects of several inflammatory cytokines in cell and animal models (Figure 1). TWEAK, for example, is a cytokine produced by several cell types (i.e., macrophages and skeletal muscle) that is capable of modulating myogenesis (Mittal et al., 2010). Initial studies demonstrated that high concentrations of exogenous TWEAK (≥100 ng/mL) in cultured myoblasts enhanced proliferation but inhibited subsequent differentiation and myotube formation (Dogra et al., 2006; Girgenrath et al., 2006). The observed impairment in cell cycle exit and muscle specific gene expression in TWEAK-treated myoblasts corresponded with decreased gene expression and protein levels of the myogenic regulatory factors MyoD and myogenin (Dogra et al., 2006; Girgenrath et al., 2006). Activation of classical (canonical) nuclear factor-kB (NF-κB) signaling appears to mediate this response. NF-κB proteins are a family of structurally similar transcription factors (p65, RelB, c-Rel, p105/p50, and p100/p52) that form either homodimers or heterodimers. Classical signaling specifically involves the phosphorylation and degradation of an inhibitory protein (IκBα) and subsequent translocation of an activated p65/p50 heterodimer to the nucleus (Bakkar and Guttridge, 2010). Inhibiting p65/p50 activity reversed the inhibitory effect of soluble TWEAK (500 ng/mL) on differentiation and MyoD protein expression in cultured myoblasts (Dogra et al., 2006), suggesting high concentrations of TWEAK limit normal myogenic progression through classical NF-κB activity.

High concentrations of TWEAK enhancing proliferation and inhibiting differentiation through classical NF-κB signaling would be critical during early phases of muscle regeneration to promote rapid proliferation and adequate expansion of the myoblast population, while simultaneously avoiding premature differentiation (Enwere et al., 2014). In contrast, TWEAK present at low concentrations (10 ng/mL) in C2C12 myoblasts has been shown to promote later stages of myogenesis through the activation of alternative (non-canonical) NF-κB signaling (Enwere et al., 2012). The alternative NF-κB signaling pathway involves the phosphorylation and partial proteasomal degradation of a p100 subunit to p52 to generate a p52/RelB heterodimer (Bakkar and Guttridge, 2010). Translocation of the p52/RelB heterodimer to the nucleus and subsequent transcriptional activity promotes myogenesis by stimulating myoblast fusion into myotubes. Exposing differentiating myoblasts to low concentrations of exogenous TWEAK (10 ng/mL) increased myoblast fusion and doubled myotube diameter when compared to untreated cells (Enwere et al., 2012).

The cytokine TNFα also regulates myogenesis. TNFα concentrations rise substantially at the site of muscle injury due to its release from injured myofibers and infiltrating immune cells (De Bleecker et al., 1999). *In vitro* work has shown that TNFα intrinsic to satellite cells and myeloid cell-derived TNFα influence the myogenic response (Wang et al., 2018). The early increase in TNFα expression functions as a chemoattractant signal that stimulates myogenic cell migration to the site of injury (Torrente et al., 2003). The initially high concentrations of TNFα also play a role in promoting proliferation (Li, 2003) and inhibiting myogenic differentiation (Langen et al., 2004). This occurs analogous to TWEAK through the activation of classical NF-κB signaling and downstream inhibition of MyoD mRNA and protein expression (Langen et al., 2004). An abundance of myeloid cell-derived TNFα also appears to reduce muscle cell fusion (Wang et al., 2018). While high concentrations of recombinant TNFα (≥0.5 ng/mL) inhibit the progression of myogenesis, low concentrations of TNFα (0.05 ng/mL) have been shown to enhance differentiation in cultured myoblasts (Chen et al., 2007). This response appears to occur through downstream activation of p38 mitogen-activated protein kinase (MAPK). Levels of activated p38 MAPK and markers of differentiation were both diminished when TNFα was neutralized in C2C12 myoblast (Chen et al., 2007). p38 activation and muscle regeneration were also impaired following cardiotoxin-injury of soleus muscle in TNFα receptor double knockout mice (p55^–/–^p75^–/–^) (Chen et al., 2005).

The cytokine IL-6 is another major regulator of myogenesis. IL-6 is secreted by infiltrating macrophages and neutrophils, FAP cells, and muscle itself (Kami and Senba, 1998; Joe et al., 2010; Zhang et al., 2013). Binding of IL-6 to its receptor activates the Janus kinase/signal transducer and activator of transcription (JAK/STAT) signaling cascade. IL-6-dependent activation of STAT3 specifically was required for satellite cell proliferation *in vitro* (Serrano et al., 2008). IL-6 is also necessary for the complete differentiation of muscle cells. Primary muscle cells from IL-6^–/–^ mice displayed a clear reduction in myotube formation indicative of decreased myoblast fusion (Hoene et al., 2013). This occurred independent of downstream STAT3 activation, although the exact mediators of this effect are unknown. How exceedingly high levels of IL-6 affect myogenesis is unclear, however, chronic overexpression of IL-6 has been shown to induce muscle wasting (Haddad et al., 2005).

In total, these findings specific to TWEAK, TNFα, and IL-6 indicate that high concentrations of these cytokines may inhibit normal myogenic progression. It is important to note, however, that the physiological relevance of these *in vitro* studies remains unclear as they generally involve acute exposure to very high concentrations of a single cytokine. The involvement of multiple cytokines under physiological conditions and the varied concentrations of cytokines between plasma and muscle interstitium due to the presence of an endothelial barrier are possible confounding factors that must be considered. Regardless, these findings collectively suggest that a transient increase in intramuscular pro-inflammatory signaling after injury is required for muscle regeneration and repair, while an excessive or persistent inflammatory response can prevent myogenesis and limit recovery.

### Inflammation Modulates Muscle Protein Turnover

**FIGURE 2:**
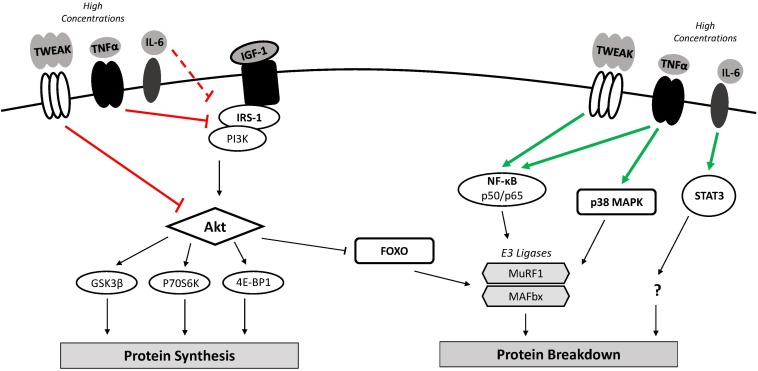
Inflammation and muscle protein turnover. Classical muscle wasting cytokines include TWEAK, TNFα, and IL-6. High concentrations of these cytokines may down-regulate mRNA translation and muscle protein synthesis through inhibition of the PI3K/Akt pathway resulting in GSK3β inhibition of translation and reduced mTOR activation. Increased FOXO nuclear localization along with classical NF-κB and p38 MAPK activity increases transcription of atrophy-related genes (i.e., MuRF1, MAFbx). An imbalance where catabolic signaling exceeds anabolic signaling can lead to muscle atrophy. 4E-BP1, 4E binding protein 1; FOXO, forkhead box O; GSK-3β, glycogen synthase kinase 3β; IGF-1, insulin-like growth factor 1; IL-6, interleukin-6; IRS-1, insulin receptor substrate 1; MAFbx, muscle atrophy F-box; mTOR, mammalian target of rapamycin; MuRF1, muscle ring finger 1; NF-κB, nuclear factor-κB; p38 MAPK, p38 mitogen-activated protein kinase; p70S6K, p70 ribosomal protein S6 kinase; PI3K, phosphoinositide 3-kinase; STAT3, signal transducer and activator of transcription 3; TNFα, tumor necrosis factor-α; TWEAK, TNF-like weak inducer of apoptosis.

Exceedingly high or chronic expression of pro-inflammatory cytokines following muscle injury impairs recovery by promoting muscle wasting (Figure 2). Differentiated C2C12 myotubes incubated with soluble TWEAK (10 ng/mL), for example, display reduced mass and a loss of total protein content (Dogra et al., 2007). Mice subjected to chronic administration of soluble TWEAK similarly exhibit reduced fiber diameter in isolated muscle sections and decreased body weight compared to control mice (Dogra et al., 2007). This effect appears to involve multiple signaling pathways that promote protein catabolism and impair anabolic signaling. Activation of NF-κB, for example, is involved in the TWEAK-induced degradation of cultured myotubes (Dogra et al., 2007). NF-κB signaling likely mediates this effect by up-regulating the ubiquitin proteasome system (UPS). The UPS is largely responsible for the degradation of myofibrillar proteins through the enzymatic activity of the muscle-specific ubiquitin ligases muscle atrophy F-box (MAFbx/Atrogin-1) and muscle ring finger 1 (MuRF1). NF-κB has been shown to regulate MuRF1 expression (Cai et al., 2004), which was increased along with MAFbx following TWEAK treatment of cultured myotubes (Dogra et al., 2007).

Inhibition of the phosphatidylinositol-3 kinase (PI3K)/Akt pathway also contributes to the observed up-regulation of UPS activity in TWEAK-treated myotubes. Active Akt phosphorylates and inhibits the forkhead box O (FoxO) transcription factor, preventing its translocation to the nucleus and induction of MAFbx and MuRF1 expression (Stitt et al., 2004). Exogenous TWEAK in cultured myotubes significantly decreased Akt and FOXO1a phosphorylation, suggesting increases in MAFbx and MuRF1 following TWEAK treatment are also due to Akt inhibition (Dogra et al., 2007). TWEAK-mediated inhibition of the PI3K/Akt is also consequential to anabolic signaling. Akt facilitates translation initiation and muscle protein synthesis through the phosphorylation and activation of the mammalian target of rapamycin (mTOR) and subsequent activity of its downstream targets, p70 ribosomal protein S6 kinase (p70S6K) and 4E binding protein 1 (4E-BP1) (Nave et al., 1999). In addition, active Akt phosphorylates glycogen synthase kinase 3β (GSK-3β), reversing its inhibitory effect on the translation initiation factor eukaryotic initiation factor 2B (eIF2B) (Jefferson et al., 1999). Phosphorylation levels of GSK-3β, mTOR, and p70S6K were decreased following TWEAK treatment of cultured myotubes, indicating impaired anabolic signaling (Dogra et al., 2007). An imbalance where catabolic signaling exceeds anabolic signaling promotes muscle wasting.

Elevated levels of TNFα modulate muscle protein turnover and induce atrophy. A dose-dependent loss of total protein was noted in differentiated skeletal muscle myotubes following prolonged exposure to TNFα (1 – 6 ng/mL) (Li and Reid, 2000). TNFα appears to mediate this effect by activating NF-κB transcriptional activity (Li and Reid, 2000) and inhibiting Akt (Sishi and Engelbrecht, 2011), triggering downstream up-regulation MAFbx and MuRF1 expression. The up-regulation of MAFbx in C2C12 myoblasts following TNFα exposure also required intact p38 MAPK, indicating the TNFα/p38 MAPK signaling axis enhances downstream UPS activity and related muscle catabolism (Li et al., 2005). Increased TNFα concentrations have also been shown to limit muscle anabolism via the PI3K/Akt signaling pathway. Upstream activation of PI3K/Akt signaling involves the binding of factors such as insulin-like growth factor 1 (IGF-1) or insulin to their receptors and the subsequent phosphorylation and activation of insulin receptor substrate 1 (IRS-1). TNFα may exert its anti-anabolic effect by downregulating IGF-1 synthesis or by direct interaction with IRS-1. Addition of TNFα to cultured myotubes decreased IGF-1 mRNA expression by 50–80% (Frost et al., 2003). TNFα has also been shown to stimulate serine residue phosphorylation of IRS-1, preventing its recruitment to the insulin/IGF-1 receptor (Hotamisligil et al., 1996). In theory, this would ultimately suppress anabolic (i.e., mTOR, p70S6K) and promote catabolic (i.e., FOXO, MuRF1, MAFbx) signaling downstream of the IGF-1 receptor.

The effects of heightened IL-6 expression on muscle anabolic and catabolic signaling pathways *in vivo* remain elusive. Locally infusing soluble IL-6 in muscles of rats significantly decreased myofibrillar protein content compared to untreated contralateral muscle (Haddad et al., 2005). Inhibiting STAT3 in C2C12 myoblasts reduced muscle atrophy downstream of IL-6, implicating the JAK/STAT3 pathway as a mediator of IL-6-induced muscle wasting (Bonetto et al., 2012). The mechanisms by which STAT3 promotes atrophy, however, are still unknown. IL-6-mediated muscle wasting may also result from a downregulation of anabolic signaling pathways. An increase in IGF-1 mRNA and decrease in downstream phosphorylation of p70S6K has been observed in IL-6-infused muscles of rats (Haddad et al., 2005). These findings suggest that IL-6 disrupts growth factor-related intracellular signaling, resulting in a compensatory increase in IGF-1 production (Haddad et al., 2005) which would potentially compromise skeletal muscle mass.

## Variability in Muscle Regenerative Capacity

Activation of satellite cells and their subsequent proliferation, differentiation, and fusion with existing myofibers is critical after muscle injury to regenerate damaged muscle tissue. However, age-related declines in progenitor cell expansion and inadequate maintenance of satellite cell quiescence impairs muscle regenerative capacity in older adults. Heterochronic parabiosis and whole muscle grafting experiments have shown that regeneration of old muscle exposed to a young environment occurs normally, implicating extrinsic factors and age-related alterations in the satellite cell microenvironment in the dysregulation of muscle regeneration with aging (Carlson and Faulkner, 1989; Conboy et al., 2005). However, recent work using isolated satellite cells has also associated cell-intrinsic defects with the age-related decline in satellite cell function suggesting regulation is multifaceted (Bernet et al., 2014; Cosgrove et al., 2014; Sousa-Victor et al., 2014).

Several molecular and cellular processes (both intrinsic and extrinsic) underlie to the loss of regenerative capacity with aging. Notch signaling in young muscle promotes proliferation of activated satellite cells, while a subsequent suppression of this pathway and transition to canonical Wnt signaling is necessary for proper differentiation and muscle growth (Conboy and Rando, 2002; Brack et al., 2008). Aging appears to disrupt the precise balance and interaction of these pathways, as Notch activity is attenuated in aged muscle (Conboy et al., 2003). Satellite cells isolated from aged mice also display high levels of Wnt signaling that attenuate progression of satellite cells through the differentiation program and promote their adoption of a fibrogenic fate (Brack et al., 2007). Recent work has suggested age-related impairments in myogensis are also tied to declines in autophagy, which causes functional deterioration of satellite cells and promotes senescence (Garcia-Prat et al., 2016). Age-related perturbations in IL-33 expression may similarly disrupt regeneration by altering the accumulation of T_reg_ cells in the satellite cell niche (Kuswanto et al., 2016). Additional changes to the satellite cell microenvironment including increased fibroblast growth factor-2 (FGF-2) expression, loss of fibronectin, systemic increases in GDF11, impaired β1-Integrin signaling, and attenuated levels of the anti-aging hormone Klotho have all been implicated in the poor repair of skeletal muscle with aging (Chakkalakal et al., 2012; Egerman et al., 2015; Lukjanenko et al., 2016; Rozo et al., 2016; Sahu et al., 2018; Welc et al., 2020).

Age-related increases in intramuscular inflammatory pathway activity may also reduce muscle regenerative potential with aging. Animal work has indicated that attenuating IL-6/JAK/STAT3 pathway activity enhances regenerative potential of aged satellite cells (Price et al., 2014). Merritt et al. (2013a) also observed differences in basal muscle pro-inflammatory signaling independent of circulating cytokines between middle-aged adults (40.4 ± 1.1 y, AGE40), older adults (61.2 ± 0.6 y, AGE61), and elderly (75.5 ± 0.7 y, AGE76) individuals. IL-6, TNFα, and TWEAK expression were higher in AGE61 and AGE76 groups relative to AGE40 at baseline and following modest muscle damage induced with a resistance exercise protocol (Merritt et al., 2013a). Myoblasts isolated from three young (28 ± 2 y) versus three older (64 ± 2 y) individuals also displayed greater inflammatory signaling in the absence of a pro-inflammatory stimulus, and had an exaggerated inflammatory response and reduced fusion capacity when treated with TNFα (Merritt et al., 2013a). This heightened basal pro-inflammatory signaling and hypersensitivity to inflammatory stimuli in individuals of advanced age was described as “muscle inflammation susceptibility,” or MuIS^(+)^. This MuIS^(+)^ phenotype, along with age-related cell-intrinsic and extrinsic defects affecting satellite cell self-renewal (Bernet et al., 2014), may impair myogenesis and promote muscle atrophy following muscle injury.

Basal levels of inflammation and the sensitivity to inflammatory stimuli may not be uniform across all populations. Follow-up work by Bamman et al. (2015) described analyses identifying the MuIS^(+)^ phenotype in certain adults across all ages, suggesting some individuals display elevated basal levels of muscle inflammation and an exaggerated or prolonged response to an inflammatory insult. This apparent interindividual variability in heightened inflammation susceptibility suggests some individuals may be more prone to an exceedingly high inflammatory response to muscle injury that would be detrimental to recovery. Bamman et al. (2015) considered this hypothesis by evaluating MuIS status in patients undergoing total hip arthroplasty (THA) for end-stage osteoarthritis, since osteoarthritis-related damage in the hip leads to immune cell infiltration and heightened inflammatory cytokine expression in the synovial fluid, synovial membrane, cartilage, and the subchondral bone layer (Wojdasiewicz et al., 2014; Mathiessen and Conaghan, 2017). In theory, evaluating MuIS status would ascertain the susceptibility of muscle surrounding the hip to the local inflammatory burden of the osteoarthritic joint (Bamman et al., 2015).

The MuIS^(+)^ phenotype was identified in a population of THA patients based on the expression of the TWEAK receptor, fibroblast growth factor inducible 14 (Fn14), in muscle surrounding the diseased hip (Bamman et al., 2015). Levels of Fn14 are generally low in healthy tissues and therefore the induction of Fn14 expression in response to injury, stress, or exercise is tied to TWEAK/Fn14 pathway activity (Enwere et al., 2014). Dichotomization into MuIS^(+)^ and MuIS^(–)^ individuals was done based on median Fn14 expression and revealed several key differences between groups. The MuIS^(+)^ group (*n* = 7) had a mean Fn14 gene expression that was five times higher than the MuIS^(–)^ group (*n* = 8) (Bamman et al., 2015). Individuals designated as MuIS^(+)^ also exhibited heightened expression of all inflammatory genes evaluated (e.g., TNFα, IL-6, TWEAK) compared to non-surgical controls, while only the up-regulation of the IL-6 receptor (IL-6R) was found in the MuIS^(–)^ group (Bamman et al., 2015). Significantly lower muscle protein synthesis (i.e., fractional synthetic rate) was observed in muscle surrounding the diseased hip in the MuIS^(+)^ versus MuIS^(–)^ groups (Bamman et al., 2015). The heightened inflammatory signaling and changes in muscle protein synthesis in the MuIS^(+)^ versus MuIS^(–)^ group would be expected to reduce myogenic activity and induce muscle atrophy. These findings suggest that dichotomization of individuals based on Fn14 expression reveals a phenotype that would be detrimental to recovery from THA, though long-term investigations evaluating the recovery potential of MuIS^(+)^ versus MuIS^(–)^ THA patients are currently lacking.

The possibility exists that these observations may not be unique to THA patients. Levinger et al. (2011) observed heightened inflammatory cytokine expression in the vastus lateralis of patients undergoing total knee arthroplasty (TKA) for end-stage osteoarthritis, suggesting the local inflammatory burden of an osteoarthritic knee joint similarly extends to surrounding musculature. Muscle inflammatory status may also predict muscle accretion and functional recovery potential following surgical repair of an acute orthopedic injury in otherwise healthy individuals. Anterior cruciate ligament (ACL) injury and reconstruction, for example, has been associated with elevated inflammatory cytokine expression in synovial fluid of the injured knee (Higuchi et al., 2006). Based on the findings in TKA patients, it is plausible that the local knee inflammation observed with ACL injury and reconstruction may similarly extend to the vastus lateralis. A heightened susceptibility to a locally inflamed joint (e.g., knee, hip, or shoulder) following musculoskeletal injury may contribute to an excessive inflammatory response to surgical treatment that limits regeneration of damaged tissue and promotes atrophy in the postoperative period. Whether this situation provides insight into a patient’s rehabilitative potential is not known.

The MuIS^(+)^ phenotype and age-related declines in regenerative capacity may have implications in the context of muscle injury given the associated muscle disuse atrophy and/or damage that demands a period of muscle growth for proper recovery. Short periods of immobilization or reduced physical activity are often necessary post-injury or postoperatively to prevent further injury and facilitate recovery. While restricting motion allows the injured area to heal, muscle loss can occur rapidly under these conditions given the decline in loading and absence of neural activation (i.e., disuse atrophy). This is evident in experimental models of disuse that have shown a ∼4% loss of quadriceps cross-sectional area after only 5 days of unilateral knee immobilization in a population of young men (Wall et al., 2016), and a ∼4% loss of leg fat-free mass after 14 days of reduced daily step count (i.e., 6000 to 1500 steps per day) in a healthy older population (Breen et al., 2013). Rehabilitation begins gradually post-injury or post-operatively to attenuate muscle losses and regain muscle mass, strength, and function in the injured limb. While muscle hypertrophy during rehabilitation is primarily mediated by periods of positive muscle protein balance (i.e., muscle protein synthesis > muscle protein breakdown) that lead to lean mass accrual when maintained over time (Phillips, 2014), myogenesis likely plays a role.

While satellite cell involvement in muscle fiber regeneration has been consistently demonstrated, the role of satellite cells in muscle fiber hypertrophy and the effects of aging are less clear. Animal studies suggest that although muscle growth can occur in satellite cell ablated conditions (2 weeks overload) (McCarthy et al., 2011), satellite cells may be necessary to support more extensive muscle fiber hypertrophy (8 weeks overload) (Fry et al., 2014). Work in humans showing the upregulation of MRFs following resistance exercise (McKay et al., 2008; Nederveen et al., 2019), and positive correlations between muscle growth and increased satellite cell content during prolonged resistance exercise training (Petrella et al., 2006; Verdijk et al., 2010; Bellamy et al., 2014), has contributed to the idea that satellite cells play a role in human muscle fiber hypertrophy (Snijders et al., 2015; Brook et al., 2019). The MuIS^(+)^ phenotype and age-related impairments in satellite cell function may therefore limit hypertrophy of skeletal muscle after an atrophy-inducing event. Suetta et al. (2013) showed that while a 4 week exercise intervention restored muscle mass lost due to 2 weeks of leg immobilization in young individuals (∼20 y), muscle fiber size was not recovered in an older population (∼70 y). Satellite cell expansion was also diminished during retraining in the old versus young individuals, suggesting an impaired regenerative response contributed to the lack of muscle restoration and repair (Suetta et al., 2013). Whether the MuIS^(+)^ phenotype impairs muscle growth after injury-related muscle atrophy has not been determined.

Potential problems with existing methods of determining MuIS status that currently limit its clinical application should be noted. Dichotomization based on Fn14 expression, for example, results in groups of individuals (i.e., above versus below the cut-off point) that are considered equal although their individual prognosis may vary considerably. Likewise, individuals close to, but on opposite sides of, the cut-off point are regarded as very different. Dichotomization based on the sample median also means that the exact value of the cut-off point could change considerably from sample to sample. Using MuIS status effectively in clinical settings would require further exploration to determine a threshold of expression of specific markers (i.e., Fn14) that would be considered detrimental to muscle recovery potential. The clinical application of this tool in its current form also has methodical limitations. Collecting a muscle sample and evaluating gene expression is outside the scope of most clinical sites and laboratories. Finally, identifying appropriate therapeutic strategies to promote functional recovery and muscle mass accretion in individuals with the MuIS^(+)^ phenotype would be necessary. Potential interventions may include more aggressive physical rehabilitation efforts (Bamman et al., 2015), or pharmacological treatment to attenuate excessive inflammation. Elucidating appropriate timing and targets for pharmacological interventions would be critical. Attenuating inflammation too early in the regeneration process may delay or diminish recovery. Likewise, targeting either pro-inflammatory or anti-inflammatory signaling, specifically, may disrupt the delicate balance of these pathways needed for proper regeneration. Treating chronic inflammation may instead require modulation of pathways involved in initiating the resolution of inflammation (Sugimoto et al., 2016). The potential benefits to determining MuIS status in orthopedic patient populations with specific regard for prognostic outcomes following surgical interventions warrants further investigation despite current limitations.

## Conclusion

Skeletal muscle has the intrinsic capacity to regenerate and repair itself following injury through myogenesis, a process regulated by a finely controlled inflammatory response. This regenerative capacity is diminished with advanced age, however, as alterations in intrinsic and extrinsic signaling regulating satellite cell activity are observed in older adults. Cell and animal models also show that while inflammatory signaling mediates the myogenic response to muscle damage, high concentrations of several inflammatory cytokines (e.g., TNFα, IL-6, TWEAK) can inhibit muscle regeneration and trigger muscle wasting. Therefore advanced age and/or exceedingly high inflammatory signaling post-injury or postoperatively may limit the repair of damaged tissue and maintenance of muscle mass. Excessive or chronic inflammation is a particular concern for a subset of individuals who display elevated levels of basal intramuscular inflammatory signaling and greater susceptibility to an inflammatory insult (i.e., MuIS^(+)^ phenotype). Future exploration of this interindividual variability in inflammatory susceptibility, focusing on understanding the cause of the observed differences, is warranted.

To date less is known about the divergent roles of inflammatory signaling following muscle injury in humans. Further investigations are needed to delineate the role of acute versus chronic inflammation as an anabolic or catabolic stimulus, respectively. Future work must also focus on molecular and cellular factors underlying the loss of regenerative capacity with aging. Understanding these processes is essential in developing clinical interventions to increase muscle regeneration and attenuate muscle atrophy after muscle injury in an effort to improve gains in muscle mass, strength, and function during rehabilitation. These interventions may also be relevant to the treatment of chronic inflammatory myopathies characterized by diminished myogenesis and muscle wasting.

## Disclosure

The views and assertions expressed herein are those of the authors and do not reflect the official policy of the Army or the Department of Defense. Any citations of commercial organization and trade names in this report do not constitute an official Department of the Army endorsement of approval of the products or services of these organization.

## Author Contributions

EH, SP, and NR conceptualized the content of the article. EH wrote the original draft. EH, SP, CB, MF, and NR reviewed, edited, and approved the final version of the manuscript.

## Conflict of Interest

The authors declare that this study received funding from the National Cattlemen’s Beef Association (NCBA) and that the funder was not involved in the study design, collection, analysis, interpretation of data, the writing of this article or the decision to submit it for publication.

## Abbreviations

4E-BP1: 4E binding protein 1
ACL: anterior cruciate ligament
AMPK: AMP-activated protein kinase
CCL2: CC-chemokine ligand 2
CXCL1: CXC-chemokine ligand 1
DAMP: damage-associated molecular pattern
Dll4: Delta-like 4
ECM: extra-cellular matrix
eIF2B: eukaryotic initiation factor 2B
FAP: fibro-adipogenic progenitor
FGF-2: fibroblast growth factor-2
Fn14: fibroblast growth factor-inducible 14
FOXO: forkhead box O
GSK-3β: glycogen synthase kinase 3β
HMGB1: high-mobility group box 1 protein
IFNγ: interferon gamma
IGF-1: insulin-like growth factor 1
IL: interleukin
IRS-1: insulin receptor substrate 1
JAK: Janus kinase
MAFbx: muscle atrophy F-box
MRF: myogenic regulatory factors
MRF4: myogenic regulatory factor 4
mTOR: mammalian target of rapamycin
MuIS: muscle inflammatory susceptibility
MuRF1: muscle ring finger 1
Myf5: myogenic factor 5
NF-κB: nuclear factor-κB
p38 MAPK: p38 mitogen-activated protein kinase
p70S6K: p70 ribosomal protein S6 kinase
Pax7: paired box 7
PI3K: phosphoinositide 3-kinase
RBP-Jk: recombining protein-Jk
STAT3: signal transducer and activator of transcription 3
Spry1: Sprouty1
TGF-β1: transforming growth factor beta 1
THA: total hip arthroplasty
TKA: total knee arthroplasty
TNFα: tumor necrosis factor-α
T_reg_: regulatory T cell
TWEAK: TNF-like weak inducer of apoptosis
Ulk1: Unc-51-like kinase 1
UPS: ubiquitin proteasome system.
